# *Cryptosporidium parvum* cyclic GMP-dependent protein kinase (PKG): An essential mediator of merozoite egress

**DOI:** 10.1016/j.molbiopara.2020.111277

**Published:** 2020-05

**Authors:** Samantha Nava, Aygul Sadiqova, Alejandro Castellanos-Gonzalez, A. Clinton White

**Affiliations:** Infectious Diseases Division, Department of Internal Medicine, University of Texas Medical Branch, 301 University Boulevard, Galveston, Texas 77555-0435, USA

**Keywords:** *Cryptosporidium*, Egress, Cyclic GMP-dependent protein kinase

## Abstract

Cryptosporidiosis is an obligate intracellular pathogen causing diarrhea. Merozoite egress is essential for infection to spread between host cells. However, the mechanisms of egress have yet to be defined. We hypothesized that Cyclic GMP-Dependent Protein Kinase G (PKG) may be involved in *Cryptosporidium* egress. In this study, *Cryptosporidium parvum* PKG was silenced by using antisense RNA sequences. PKG-silencing significantly inhibited egress of merozoites from infected HCT-8 cells into the supernatant and led to retention of intracellular forms within the host cells. This data identifies PKG as a key mediator of merozoite egress, a key step in the parasite lifecycle.

## Highlights

• Cryptosporidium protein kinase G mRNA was silenced using siRNA, which led to decreased expression of the PKG protein.
• After silencing, merozoite egress was blocked and merozoites retained within the host epithelical cells.
• PKG plays an essential role in Cryptosporidium merozoite egress.

## Graphical abstract

Silencing Cryptosporidium Protein kinase G blocks merozoite egress

**Figure:**
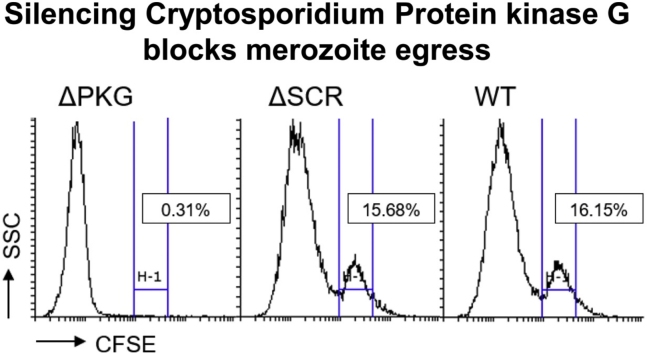

Cryptosporidiosis is a disease that affects human and animal populations throughout the world. Human cryptosporidiosis presents as a gastrointestinal infection that can be a severe and life-threatening disease in young children and immunocompromised patients. *Cryptosporidium* is a major cause of diarrheal disease in children during the first two years of life [[1], [2], [3], [4]]. Recurrent infections are associated with malnutrition and impaired physical and cognitive development in children [3]. Nitazoxanide is the only FDA-approved drug to treat cryptosporidiosis, but its efficacy is limited in patients with AIDS and other high-risk groups [4,5].

Study of cryptosporidiosis has been hampered by an incomplete understanding of the molecular mechanisms involved in the parasite lifecycle. Cryptosporidiosis is transmitted by hardy oocysts, which release the invasive sporozoites. The sporozoites invade the host intestinal epithelial cells forming parasitophorous vacuoles inside the host membrane, but separated from the cell cytoplasm. Within the parasitophorous vacuole, the parasite proliferates, forming meronts and merozoites. The merozoites egress from the infected cells and re-invade other epithelial cells. Thus, egress is a key event for the completion of the life cycle that leads to propagation of the parasite and establishment of infection. However, little is known concerning the molecular mechanisms behind egress in *Cryptosporidium*.

Cyclic-GMP dependent protein kinase G (PKG) has been identified in related organisms as a key mediator of the signal transduction cascade leading to merozoite egress [[6], [7], [8], [9], [10]]. In *Toxoplasma gondii*, PKG is upstream of microneme secretion and a cofactor in subsequent egress [[9], [10], [11]]. In *Plasmodium falciparum*, inhibition of PKG activity has been found to block egress, leading to clearance of infection and decreased transmission to mosquitoes [7,12]. We identified a homologue of PKG in the *C. parvum* genome. In this study, we used a recently developed RNA silencing method to test the hypothesis that PKG is an essential mediator of egress of *Cryptosporidium* merozoites from host cells [13].

To determine the time course of PKG expression, we used human colonic epithelial cells (HCT-8; ATCC, Manassas, VA) for in vitro infection. HCT-8 cells were grown overnight (at 37 °C 5% CO_2_) in 24-well plates (Corning, Tewksbury, MA) until ∼80 % confluent. Viable *Cryptosporidium* oocysts were excysted using 60 μl acidic water (pH ∼2.5, 10 min on ice) followed by 250 μl of 0.8 % taurocholate in RPMI media (37 °C for 1 h). Release of sporozoites was confirmed by light microscope and sporozoites were separated from unexcysted oocysts using a 3 μm-pore sized filter (Millipore Sigma, Burlington, MA). Sporozoites were then incubated with HCT-8 monolayers (2 h, 37 °C, 5% CO_2_) to establish basal infection. Monolayers were gently washed with ∼100 μl PBS once to remove parasites that had not entered host cells and then incubated with 300 μl RPMI 10 % fetal bovine serum (37 °C, 5% CO_2,_ 26 h). The timing of merozoite egress was determined as previously described [14]. To determine the time course of PKG expression, infected monolayer and supernatant were lysed directly from culture plates by adding 350 μl of Lysis buffer from Qiagen RNeasy Kit (Qiagen, Valencia, CA) at 1, 2, then every 2 h through 26 h post infection. Lysate was stored at −20 °C until total RNA was extracted. PKG expression was measured by qRT-PCR using 20 ng of RNA. The following conditions were used for amplification: 55 °C for 2 min, 95 °C for 5 min, and 40 cycles at 95 °C for 15 s and 65 °C for 1 min using an AB7500 Fast PCR System for 98-well plates (Life Technologies; Grand Island, NY). Primers are listed in Supplementary Table 1. All samples were analyzed in triplicate in at least two independent experiments.

Antisense sequences with a length of 19–21 nucleotides were designed complimentary to PKG (cgd8_750; XM_626985.1) [13]. To assemble the silencer complex, 250 ng of purified human Ago2 (Sino biological, China) was incubated with 1 μM of antisense RNA (5′ **AUA UCU UGU GAA AGA AUA AAG CUA** 3′) and 15 μl of assembly buffer (39 mM HEPES, pH 7.4, 150 mM potassium acetate, and 2 mM magnesium chloride (MgCl_2_) for 1 h at room temperature (RT) [13,15]. For parasite transfection, first 10 μl of lipid-based transfection reagent (Pierce Protein Transfection Reagent, Thermo Fisher Scientific, Waltham, MA) was incubated with the silencer complex at RT for 30 min. Next, 5 × 10^5^ oocysts were incubated with the mixture for 2 h at room temperature followed by incubation for 2 h at 37 °C. To confirm silencing, ∼20 ng of total RNA was extracted from the oocysts using the Qiagen RNeasy kit (Qiagen, Valencia, CA) and stored at −20 °C until further analysis by real-time qPCR as described above. Silencing potency was determined in three independent experiments, each with biological duplicates, and technical triplicates.

With wild-type parasites in our system, merozoite numbers peak in the supernatant at 24 h post infection [14]. To measure merozoite egress, supernatants were collected from samples at 1, 20, 24, and 26 h post-infection and filtered (3 μm pore-size) to separate merozoites from detached host cells. Samples were incubated in 350 μl of lysis buffer (RLT buffer; RNeasy RNA extraction kit, Qiagen, Valencia, CA) and stored at −20 °C until total RNA was extracted. Parasite number was determined by amplifying parasite RNA lactate dehydrogenase (LDH) using qRT-PCR and compared to a standard curve prepared from mRNA extracted from defined numbers of sporozoites using conditions noted above. Primers are listed in Supplementary Table 1. All samples were analyzed in triplicate in at least two independent experiments.

We used confocal microscopy to visualize *Cryptosporidium* merozoites trapped in the host cells when PKG expression was silenced. HCT-8 cells were grown on 22 × 22 mm (1.5 mm thick) coverslips until confluency was reached. We infected human cells with approximately 5 × 10^5^ oocysts that were excysted and separated from empty shells with a filter. Parasites were silenced with antisense-PKG (ΔPKG) or wild-type controls. Infection was carried out for 24 h, 37 °C, 5%CO_2_. After infection, supernatants were removed, and infected cells were gently washed once with PBS. 4% paraformaldehyde was added to fix the cells and incubated for 25 min at RT. We treated cells with 100 μl of permeabilization buffer (0.1 % Triton x-100 in PBS) for 5 min and washed 3 times with 100 μl PBS. Approximately 100 μl antifade mounting medium with DAPI (Vectashield, Burlingame, CA) was used to stain nuclei of host cells and parasites. A coverslip was added to samples and dried/stored at 4 °C until use. Samples were then visualized with a confocal microscope (LSM 880 with Airyscan, Zeiss, Oberkochen, Germany).

Fluorescent microscopy was used to determine the viability of sporozoites after silencing. For these experiments, we silenced PKG in *Cryptosporidium* oocysts, as described above, then added 2 μl of 0.5 μM of carboxyfluorescein succinimidyl ester (CFSE), which is cleaved to the fluorescent product in live cells, and incubated samples at 37 °C for 30 min. After staining, oocysts were washed once with ∼100 μl PBS and then excystation induced using acidic water followed by taurocholate as described above. Sporozoites were separated from unexcysted oocysts with a 3 μm pore-sized filter and visualized using a fluorescent microscope (Nikon 80i; Nikon, Edgewood, NY). Presence of fluorescent sporozoites indicated viable parasite that had undergone excystation.

To quantify merozoite egress to supernatants, ΔPKG or wild-type parasites were stained with CFSE prior to infection of HCT-8 cells as described above. We collect ∼ 300 μl supernatant at 1, 22, 24, and 26 h post-infection and separated merozoites from host cells and debris by filtration (3 μm pore size). Samples were fixed with 200 μl of 2% paraformaldehyde (10 min on ice) and 250 μl of PBS was added for volume. Stained parasites were acquired using a Stratedigm SE500 Analyzer (Stratedigm; San Jose, CA). Viable (stained) merozoites were quantified using FACS side-scatter gating on CFSE positive cells.

When *Cryptosporidium* sporozoites infected HCT-8 cells, PKG mRNA peaked at 18−20 hours post-infection (Fig. 1A). This peak expression of PKG mRNA preceded egress by a few hours as would be expected for molecules leading to egress. We tested potency of guide sequences to silence PKG expression in oocysts. Δ-PKG parasites exhibited a 98–99 % reduction in PKG mRNA in comparison to wild-type samples (p < 0.0001, Fig. 1B). Results were representative of five independent experiments with consistent results. To control for non-specific effects of the transfection, we used a scrambled ssRNA complexed to Ago2 and found the PKG mRNA levels similar to wild-type (Fig. 1B). To confirm that loss of PKG did not affect parasite viability, we induced excystation and determined viability with the vital dye CFSE. In comparison to wild-type and scrambled-ssRNA-Ago2 controls, Δ-PKG oocysts did not have a reduction in excystation or CFSE staining. Thus, decrease of PKG expression in *C. parvum* oocysts, did not affect viability or excystation of the parasites.

**Fig. 1:**
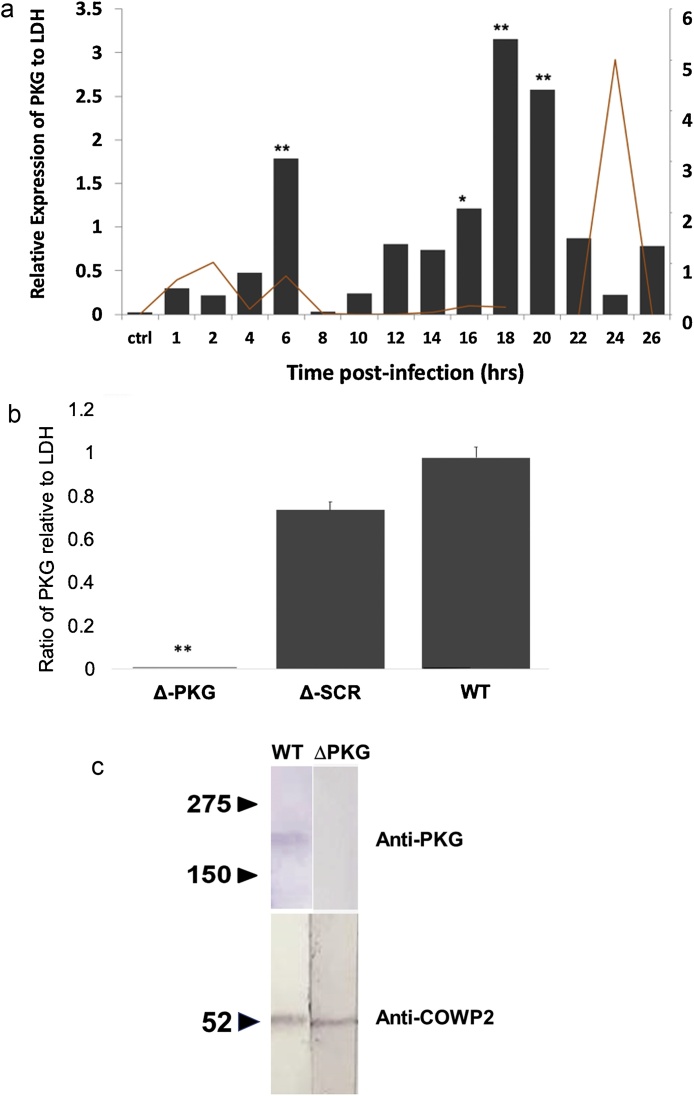
Timing of expression of PKG and merozoite egress; Silencing of parasite protein kinas G. A) Expression pattern of PKG post-infection and time point of egress of *Cryptosporidium* merozoites into supernatants. Infectious *Cryptosporidium* sporozoites invaded confluent HCT-8 cells plated on 24-well plates. PKG expression was measured in infected cells by rtPCR using PKG primers relative to a parasite housekeeping gene (LDH). PKG had a small peak at 6 -h post-infection and peak expression at 18-20 h post-infection (***p < 0.0001, *p < 0.01). This is a representative experiment, which was repeated 4 times with similar results. Parasite-LDH was measured by reverse transcription qPCR relative to a standard curve of *C. parvum* sporozoites. **B) Silencing of parasite Protein-kinase G**. Antisense sequences incubated with recombinant human Argonaute and transfected into oocysts. The ratio of parasite PKG to LDH mRNA was markedly reduced by transfection with a PKG-specific ssRNA bound to Argonaute compared to scrambled-antisense and wild-type controls (∼99 %) (***p < 0.0001). **C) PKG detection by Western Blotting.** Crude protein extracts from wild type (WT) and silenced parasites (□PKG) were transferred to PVDF membranes and tested by western blot using using antbody to PKG or Cryptosporidium outer wall protein 3 as a control.

We evaluated PKG protein levels in silenced (ΔPKG) and wild type (WT) parasites by western blotting. For these experiments we used ∼5 × 10^5^ oocysts per sample. We induced the silencing of PKG (ΔPKG) as described before. Oocysts were concentrated by centrifugation by centrifugation, resuspended in 50 μL of RIPA lysis buffer (Thermo Scientific, Waltham, MA) and incubated at 95 C for 15 min. with frequent vortexing. The crude protein extract was stored at -20 °C until use. Five μl of crude extract was mixed with 5 μL of Laemmli Sample Buffer with 1 μL of 2-Mercaptoethanol (2 min at 95 °C), then proteins were separated by SDS-PAGE electrophoresis, transferred to polyvinylidene difluoride (PVDF) membranes. After blocking the membranes overnight, with tris-buffered saline containing 0.1 % Tween and 3% powdered milk, Rabbit polyclonal antibody to human PKG (ENZO, Farmingdale NY, diluted 1:100) [16] or antibody to *Cryptosporidium* COWP2 (BEI Resources, Manassas, VA, diluted 1:1000). was added and incubated for 3 h at room temperature. Specific proteins were detected with an alkaline phosphatase conjugated antibody to rabbit IgG (Biorad, Hercules, CA).. We demonstrated expression of PKG in wild type parasites, but absence of expression in ΔPKG parasites. However, there was no decrease in expression of the parasite protein COWP2 (Fig. 1C).

To determine the effect of PKG on egress, we infected HCT-8 cells with Δ-PKG or wild-type parasites. At 24 h post-infection, Δ-PKG parasites expressed a significant decrease in merozoites mRNA released into the supernatant (p < 0.05, Fig. 2A). At 1 h and 20 h post-infection, there were minimal levels of parasites in supernatant in both Δ-PKG and wild-type samples. However, the wild-type parasites exhibited a peak of merozoite release at about 24 h post-infection, which decreased with subsequent reinvasion. By contrast, the Δ-PKG parasites did not exhibit a peak at any of the time points. However, testing the residual monolayer demonstrated significantly increased numbers of parasites (Fig. 2C). To further confirm our results, we stained Δ-PKG parasites with CFSE prior to infection and quantified parasites released from host cells by flow cytometry. Merozoites were identified by size and fluorescence as previously described [14]. Wild-type parasites and parasites silenced with scrambled-ssRNA-Ago2 had similar percentages of merozoites within the total population collected (15.7 % and 16.2 %, respectively). However, for Δ-PKG samples only 0.35 % of cells were CFSE positive and within the merozoite gate (p < 0.01. Fig. 2B). Both controls were significantly higher than Δ-PKG samples. Using confocal microscopy with DAPI staining, we demonstrated an increased number of parasites (identified by size and morphology) retained within host cells compared to wild-type and scrambled-ssRNA controls (Fig. 2D).

**Fig. 2:**
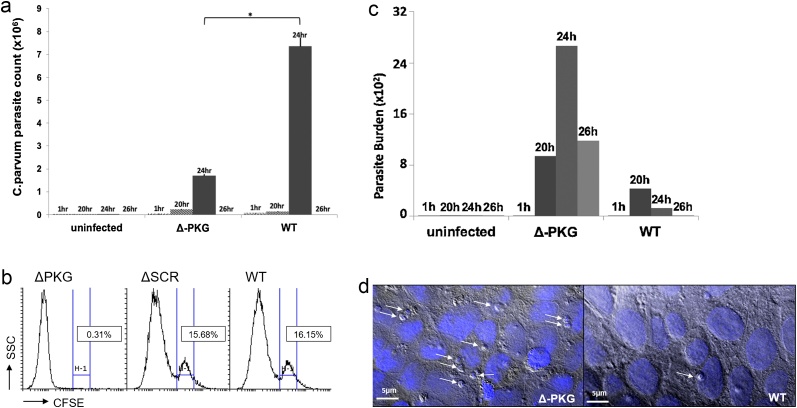
Merozoite egress in Δ-PKG parasites. A) Parasite count demonstrated in supernatant of timepoints surrounding the timepoint of egress by qPCR. The peak of egress at 24 h post-infection was significantly decreased in Δ-PKG compared to in wild-type parasites. Furthermore, egress is neither delayed nor advanced in silenced parasites as noted at 20 and 26 h post-infection, which display similar numbers to wild-type samples (p < 0.05). **B)** Sporozoites were stained with CFSE prior to infection. At 24 h post-infection, merozoites in the supernatant were quantified using flow cytometry. Scrambled-ssRNA-Ago-2 and wild-type controls showed similar levels of parasites (15.68 % and 16.15 %, respectively). However, Δ-PKG were significantly reduced (0.31 %). This experiment is representative of three independent experiments (p < 0.05). **C)** HCT-8 cells infected with Δ-PKG and WT type parasites. At 24 h post-infection, greater concentrations of parasites are found inside of host cells infected with Δ-PKG parasites compared to wild-type samples. **D)** Infected HCT-8 cells were stained with DAPI and visualized with confocal microscopy. Parasites were identified by their smaller size and location. ΔPKG-*C. parvum* parasites demonstrated higher levels of trapped parasites within host cells compared to wild-type or scrambled controls. Scale bar represents 5 μm.

Merozoite egress is an important event that contributes to the completion of the *Cryptosporidium* life cycle. The parasites develop in a vacuole that is separated from the host cell cytoplasm. Thus, unlike many other intracellular pathogens, there is no direct spread from cell to cell. Instead egress is essential for proliferations and development of disease. Here, we demonstrated that peak expression of PKG preceded egress. PKG has been implicated as an essential trigger for egress of other apicomplexans. For example, *Toxoplasma gondii* PKG has been identified as a key regulator of tachyzoite egress in coordination with calcium-dependent protein kinases (CDPKs) [6,9,10]. Loss of *Plasmodium falciparum* PKG has been associated with inhibition of parasite growth and reduction in transmission to mosquitoes [12]. *P. falciparum* PKG is upstream of a signaling cascade involving subtilisin-like serine protease (SUB1) and serine repeat antigens (SERA), which degrade the parasitophorous vacuolar membrane upon activation [7,17]. We also demonstrated that silencing of *Cryptosporidium* SUB1 inhibited merozoite release and that SUB1 expression peaked prior to egress, consistent with packaging in vesicles for subsequent secretion [14]. Interestingly, in the malaria model, PKG was also crucial for invasion. Based on these studies, we hypothesized that the PKG homologue in *C. parvum* would play a vital role in regulation of merozoite egress and subsequent completion of the life cycle.

We then demonstrated that silencing of PKG significantly affected the ability of merozoites to egress from host cells (Fig. 2A). Additionally, we collected supernatants before and after the typical timing of egress and confirmed that egress was blocked, rather than advanced or delayed. Furthermore, we confirmed these results via flow cytometry of supernatants and confocal microscopy of the residual cell layer (Fig. 2B, D).

The molecular mechanisms of egress vary between apicomplexans. The work from this study demonstrates that PKG is crucial for *Cryptosporidium* merozoite egress as has previously been observed for *Plasmodium* and *Toxoplasma*. In contrast to *Toxoplasma*, CDPK did not seem to play a role in egress [14]. Similar to *Plasmodium*, however, egress requires subtilisin-like proteases [14]. These observations may suggest a model of egress similar to that observed in *Plasmodium*, however further studies will need to be carried out to confirm this theory.

Egress marks a dramatic event within the parasite lifecycle that causes host cell death and leads to the spread of infection to additional host cells. It is a parasite-driven process that is not well understood in *Cryptosporidium*. In related organisms, PKG has been targeted for the development of novel chemotherapeutic agents that show promising results [6,12]. As a homologue of these targets, PKG in *C. parvum* was identified as a necessary component for egress. Further studies are needed to further elucidate the mechanism behind egress in this parasite. However, these results are the initial insight to the signaling activity that could contribute to the development of therapeutics for cryptosporidiosis.

## CRediT authorship contribution statement

**Samantha Nava:** Conceptualization, Methodology, Formal analysis, Writing - original draft. **Aygul Sadiqova:** Writing - review & editing, Methodology. **Alejandro Castellanos-Gonzalez:** Conceptualization, Methodology, Supervision, Writing - review & editing. **A. Clinton White:** Conceptualization, Supervision.
